# Left atrial appendage aneurysm resection following the onset of a cardiogenic stroke: a case report

**DOI:** 10.1093/jscr/rjac549

**Published:** 2022-11-28

**Authors:** Hideki Isa, Tomoki Nakatsu, Fumiaki Kimura

**Affiliations:** Department of Cardiovascular Surgery, Kushiro Kojinkai Memorial Hospital, Aikoku 191-212, Kushiro 085-0062, Hokkaido, Japan; Department of Cardiovascular Surgery, Kushiro Kojinkai Memorial Hospital, Aikoku 191-212, Kushiro 085-0062, Hokkaido, Japan; Department of Cardiovascular Surgery, Kushiro Kojinkai Memorial Hospital, Aikoku 191-212, Kushiro 085-0062, Hokkaido, Japan

## Abstract

A left atrial appendage aneurysm (LAAA) is a rare congenital or acquired anomaly that often causes fatal complications. Although many reports recommend surgical resection for treatment, there is no clear definition of LAAA. Therefore, the diagnosis and treatment are ambiguous. A 73-year-old woman with cardiogenic stroke was admitted to our hospital because of a suspected LAAA as the source of the embolus. She was incidentally diagnosed with LAAA seven years ago, which was managed with continuous anticoagulation therapy, although atrial fibrillation was not observed. The patient underwent aneurysm resection, and the postoperative course was uneventful.As LAAA symptoms are nonspecific, careful observation is required when LAAA is suspected. The risks associated with surgery are generally low and the surgical outcome is good; however, even with appropriate medical therapy, fatal complications can occur. Therefore, surgical resection of the LAAA should be considered even in asymptomatic patients, considering the low surgical risk.

## INTRODUCTION

A left atrial appendage aneurysm (LAAA) is a rare congenital or acquired anomaly that often causes fatal complications. Although many studies recommend surgical resection for treatment, there is no clear definition of LAAA; therefore, diagnosis and treatment are often ambiguous. Herein, we report a case in which LAAA was detected incidentally and followed up for seven years with anticoagulation therapy; however, surgery was eventually performed because of the onset of stroke.

## CASE REPORT

**Figure 1:**
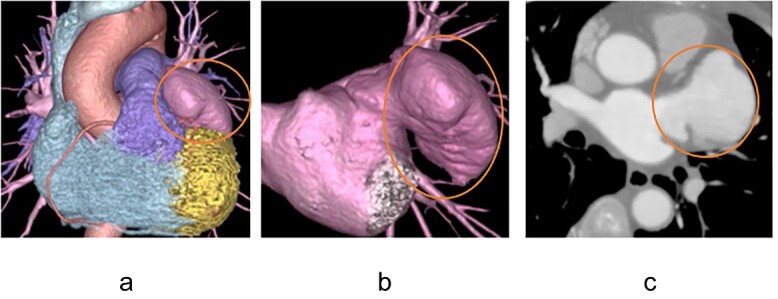
Preoperative contrast-enhanced computed tomography image A large left atrial appendage aneurysm was found, but no internal thrombus is visible.

A 73-year-old woman with cardiogenic stroke was admitted to our department for surgery because of a suspected LAAA as the source of the embolus. The LAAA was detected incidentally on coronary computed tomography (CT) angiography seven years ago, but she was followed up with direct oral anticoagulants (DOAC) to prevent thrombus formation, although atrial fibrillation (AF) was not observed. Physical examination results were unremarkable. Chest X-ray (CXR) film showed enlargement of the left atrium appendage. Transthoracic echocardiography (TTE) revealed an echo-free space connecting the left atrium adjacent to the posterolateral wall of the left ventricle. There was no thrombus in the visible range and the left ventricular ejection fraction and valve function were normal. Preoperative CT showed a huge LAAA that remained unchanged for seven years (Fig. 1). Holter electrocardiograms were taken seven times before the surgery, but AF was not observed; therefore, the operative plan was to perform LAAA resection only.

**Figure 2:**
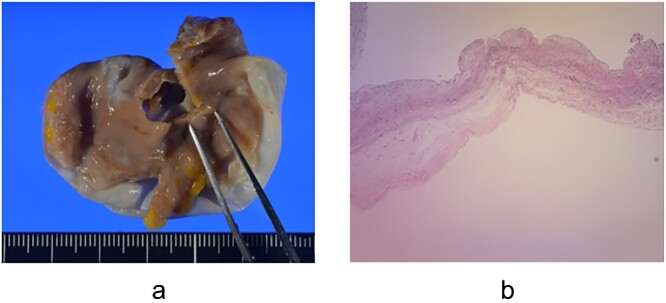
Resected left atrial appendage aneurysm and pathological specimen **(a)** The size of the left atrial appendage aneurysm was 5.5 × 4.0 × 1.5 cm, and no thrombus was observed inside; **(b)** Pathological examination showed preserved but thinning myocardial tissue and mucous degeneration.

The patient underwent median sternotomy. Cardiopulmonary bypass (CPB) was established via the ascending aorta and superior and inferior vena cava. AF occurred during CPB establishment; we then decided to perform pulmonary vein isolation (PVI). The right upper and lower pulmonary veins were detached and right-sided PVI was performed using CryoICE (AtriCure, Inc., Cincinnati, Ohio, USA). The aorta was clamped and antegrade cold blood cardioplegia was administered. Left-sided PVI was then performed in the same manner. The LAAA was resected, and the resection stump was double-sutured closed with 4–0 Prolene. The size of the LAAA was 5.5 × 4.0 × 1.5 cm and no thrombus was observed inside (Fig. 2a). The aorta was de-clamped, the heartbeat resumed spontaneously, and surgery was completed. The postoperative course was uneventful and the patient was discharged unaided on postoperative day 10. Pathological examination revealed preserved but thinning myocardial tissue and mucous degeneration (Fig. 2b). She continues to take a DOAC after surgery as a precaution but has been in sinus rhythm.

## DISCUSSION

While some LAAA patients are asymptomatic, others may present with symptoms including palpitation, chest pain, dyspnea on exertion, and embolic disorders of cerebral circulation due to thrombus formation, arrhythmias, or compression of coronary arteries [1]. As these symptoms are nonspecific, LAAA is often detected incidentally during testing. TTE is widely used because it is noninvasive, easy to perform, and contributes to the detection of LAAA, but its sensitivity is not high (45%) [2]; therefore, additional tests such as CT or transesophageal echocardiography are necessary when TTE shows findings suspicious for the presence of LAAA. In this case, LAAA was detected on CT taken during examination for chest pain. However, if the patient is asymptomatic, routine CXR may be the only opportunity to detect it; therefore, careful observation is necessary.

In this case, considering the high risk of stroke, surgery was performed via a median sternotomy with CPB, and PVI was added as AF occurred. Other surgical techniques include endoscopic resection and left thoracotomy. Endoscopic techniques can make the procedure less invasive and may be an option for small LAAA with no thrombi [3]. Regarding intervention for atrial arrhythmia, some argue that intervention is unnecessary since the arrhythmia disappears when the aneurysm is removed [4], while others argue that intervention is necessary because it may not be possible to entirely remove the dysmorphic atrial tissue that causes the arrhythmia [5]. Although controversial, there is a report of atrial flutter after LAAA resection requiring catheter ablation [6]; thus intervention for arrhythmia may be considered. The full-maze procedure is more likely to achieve sinus rhythm; however, it prolongs the operative time. In addition, intraoperative AF was paroxysmal; therefore, only PVI was performed. AF has not been observed since surgery, but careful follow-up is required.

In cases of intracardiac thrombus, surgery may be delayed, allowing anticoagulation to resolve the thrombus [2]. However, since the risks associated with surgery are generally low and the surgical outcome is good, surgical resection is recommended when LAAA is detected, even in asymptomatic patients [1]. In this case, after seven years of follow-up following the detection of LAAA, the patient underwent surgery because of the onset of stroke. The fact that the patient eventually developed a stroke despite anticoagulation therapy during follow-up suggests that earlier surgical intervention was necessary. At present, there is no clear definition of LAAA, which makes the diagnosis difficult. It is reported that an LAAA can be defined as a left atrial appendage with the diameter of the orifice, body width, and length larger than 2.7 cm, 4.8 cm, and 6.75 cm, respectively [2], which can be used as one indicator. For patients who cannot undergo surgery for various reasons, medical therapy for arrhythmias and thrombosis is necessary. However, as in this case, surgical resection is strongly recommended since complications can occur even with appropriate treatment. In this case, LAAA resection was performed because of the onset of stroke seven years after the detection of LAAA. Some LAAA patients are asymptomatic, while others may present with nonspecific symptoms; therefore, when LAAA is suspected on CXR or TTE, careful observation and additional testing are necessary. Although medical therapy is sometimes preferred, early surgical resection, including intervention for arrhythmias, should be considered. LAAA can cause fatal complications even with appropriate treatment, and the risks associated with surgery are generally low with a good surgical outcome.

## CONSENT FOR PUBLICATION

Written informed consent was obtained from the patient for scientific activities, including the publication of this report.

## Data Availability

Data supporting the conclusions are included in the article.
